# Cluster of Symptomatic Graft-to-Host Transmission of Herpes Simplex Virus Type 1 in an Endothelial Keratoplasty Setting

**DOI:** 10.1016/j.xops.2021.100051

**Published:** 2021-08-12

**Authors:** José Afonso Guerra-Assunção, Jeroen J.A. van Kampen, Sunando Roy, Lies Remeijer, Judy Breuer, Georges M.G. M. Verjans

**Affiliations:** 1 Infection and Immunity Department, University College London, London, United Kingdom; 2 Department of Viroscience, Erasmus Medical Center, Rotterdam, The Netherlands; 3 Rotterdam Eye Hospital, Rotterdam, The Netherlands

**Keywords:** Cornea bank, Cross-contamination, Descemet's membrane endothelial keratoplasty, Graft-to-host transmission, Herpes simplex virus type 1, Whole viral genome sequencing, AC, anterior chamber, DMEK, Descemet's membrane endothelial keratoplasty, FECD, Fuchs’ endothelial corneal dystrophy, HSV-1, herpes simplex virus type 1, KP, keratic precipitate, PCR, polymerase chain reaction, PKP, penetrating keratoplasty, RT-PCR, real-time polymerase chain reaction

## Abstract

**Purpose:**

Descemet's membrane endothelial keratoplasty (DMEK) is becoming the gold standard to treat corneal endothelial dysfunctions worldwide. Compared with conventional penetrating keratoplasty, infectious complications after DMEK are ill defined. We describe the clinical picture of 2 DMEK recipients, operated on the same day and in the same clinic, who developed atypical herpes simplex virus type 1 (HSV-1) infection in the transplant recipient eye within days post-DMEK. Because recipients received cornea tissue from 2 different donors prepared by the same eye bank, the likelihood of a common HSV-1 source was determined.

**Design:**

Case series.

**Participants:**

Two DMEK recipients who developed atypical intraocular HSV-1 disease shortly after surgery and surplus cornea specimens of 6 donors.

**Methods:**

Surplus cornea donor (pre-DMEK cornea remnants and conditioned cornea storage and transport media) and recipient samples (post-DMEK aqueous humor) were assayed for HSV-1 DNA and infectious virus by real-time polymerase chain reaction (RT-PCR) and cell culture, respectively. Target-enriched whole viral genome sequencing was performed on HSV-1 DNA–positive ocular specimens.

**Main Outcome Measures:**

Clinical picture of atypical intraocular HSV-1 infection post-DMEK and presence and homology of HSV-1 genomes between ocular specimens of DMEK donors and recipients.

**Results:**

Herpes simplex virus type 1 DNA was detected in aqueous humor and donor cornea specimens of both DMEK cases, but not in the cornea remnants of 6 randomly selected donors processed by the same eye bank. Infectious HSV-1 was isolated from the cornea remnant and corresponding culture medium of 1 cornea donor. Notably, whole-genome sequencing of virus DNA-positive specimens demonstrated exceptionally high genetic similarity between HSV-1 strains in recipient and donor specimens of both DMEK cases.

**Conclusions:**

Data indicate cross-contamination of cornea grafts during DMEK preparation with subsequent graft-to-host HSV-1 transmission that caused atypical sight-threatening herpetic eye disease shortly after DMEK. Ophthalmologists should be aware that HSV-1 transmission by DMEK is possible and can lead to atypical ocular disease, a condition that can easily be prevented by taking appropriate technical and clinical measures at both eye bank and surgical levels.

The cornea is the most commonly transplanted tissue worldwide.^1^ During the past 2 decades, significant advances have been made in corneal transplantation techniques. Treatment of corneal endothelial dysfunctions has evolved from replacement of a full-thickness cornea, known as penetrating keratoplasty (PKP), to replacement of only the affected cornea layer, thereby lowering allograft rejection and enabling faster visual recovery and less astigmatism.^1^^,^^2^ In 2019, more than 30 000 lamellar corneal transplantations were performed in the United States, accounting for more than 60% of annual domestic keratoplasties.^3^ Descemet's membrane endothelial keratoplasty (DMEK) involves the selective replacement of diseased corneal endothelium and Descemet’s membrane with donor tissue.^1^^,^^2^^,^^4^ Descemet's membrane endothelial keratoplasty is becoming the gold standard for treatment of corneal endothelial dysfunction, especially in Fuchs’ endothelial corneal dystrophy (FECD), which is the most common indication for keratoplasty worldwide (39%).^5^^,^^6^

Any type of corneal grafting is associated with risk of transmission of infectious agents resulting in keratitis, endophthalmitis, or even systemic infection.^1^^,^^6^^,^^7^ Presence of a corneal lamellar interface between recipient and donor tissue adds specific postoperative complications, including graft detachment and occasionally interface infections that are usually of bacterial or fungal origin.^8^^,^^9^ Herpesviruses, especially herpes simplex virus (HSV) and varicella-zoster virus, can cause a variety of corneal diseases in PKP recipients. The majority of cases are due to reactivation of latent virus, but occasionally graft-to-host HSV transmission occurs.^10^, ^11^, ^12^, ^13^ The low prevalence of herpesvirus DNA in the corneoscleral rims of donor corneas used for PKP supports these observations.^14^^,^^15^ Compared with PKP, virus-induced ocular complications in lamellar keratoplasty recipients are ill defined.^7^, ^8^, ^9^, ^10^

In this study, we determined the origin of HSV-1 that was detected in the anterior chamber (AC) taps of 2 FECD patients who developed an atypical inflammatory reaction in the anterior segment of the eye within days post-DMEK, which were performed on the same day and in the same cornea transplant setting.

## Methods

### Diagnostic Virology

Herpes simplex virus type 1 seroprevalence was determined on serum by enzyme-linked immunosorbent assay (Zeus Scientific). Virus culture and DNA extraction from clinical specimens and subsequent HSV-1–specific real-time polymerase chain reaction (RT-PCR) were performed as described.^14^ The study was performed according to the principles outlined in the Declaration of Helsinki and was approved by the local ethical committee (METC-2015-306). Written informed consent was obtained from patients 1 and 2, but patient 3 was lost to follow-up.

### DNA Extraction, Library Construction, Targeted Enrichment, and Sequencing

Total DNA was extracted from each sample by using a QiaAMP DNA minikit (Qiagen) according to the manufacturer's instructions. DNA quantification was performed with a NanoDrop spectrophotometer. Whole-genome amplification using GenomiPhi V2 (GE Healthcare) was performed using 10 ng of starting material. Libraries were constructed in accordance with the standard SureSelect XT v1.5 protocols (Agilent). Enrichment for HSV sequences was performed as described previously.^16^^,^^17^ Sequence libraries were multiplexed and sequenced using 2×250-base pair paired-end kits on an Illumina MiSeq sequencer.

### Genome Assembly and Variant Calling and Alignment

Raw sequence data were quality assessed and trimmed using fastp.^18^ Reads mapping to the human genome were assessed and removed using the kraken2 metagenomics pipeline.^19^ Nonhuman reads were mapped to the optimal RefSeq reference sequence (GenBank ID: NC_001806.2) using BWA-MEM.^20^ To improve mapping and avoid considering certain regions of the genome multiple times, terminal repeat regions were removed.^21^ We compared reference-based variant calling, including single nucleotide polymorphisms and small insertions and deletions, followed by consensus building using BCFTOOLS and de novo assembly analysis using the VIPR pipeline (Supplementary Fig 2).^22^^,^^23^ Both approaches were used to reduce the dependency of our conclusions on the methodological choices, as well as to account for the wide variation in sequencing depth between samples in this study. Consensus sequences, contiguated de novo assemblies, and publicly available sequences were aligned using the G-INS-I algorithm implemented in the MAFFT software and a maximum likelihood phylogenetic tree computed using IQ-TREE.^24^^,^^25^ Distance analyses were done using the ape package.^26^ Sample phylogenetic networks were generated using Population Analysis with Reticulate Trees.^27^ Recombination assessment was performed using RDP4.^28^

### Data Availability

Sequence data generated for all samples in the present study are available in the European Nucleotide Archive under the following accession numbers: ERX5039984, ERX5039994, ERX5040005, ERX5040027, ERX5039882, ERX5039979, ERX5039986, ERX5039998, ERX5040008, ERX5040036, ERX5039988, and ERX5040001 (Table S1).

## Results

### Patients

Patient 1, a 74-year-old man, underwent DMEK surgery in his left eye to restore endothelial function due to FECD. He developed high intraocular pressure and ocular inflammation 5 days post-DMEK, affecting not only the graft but also the recipient corneal tissue. Diffuse whitish keratic precipitates (KPs) were noted that were nonresponsive to subconjunctival steroids. No signs of bacterial or fungal infection were detected, and the clinical picture was not suspect for endophthalmitis. Diagnostic AC tap performed 10 days post-DMEK demonstrated HSV-1 DNA in the affected eye by RT-PCR (Table 1). The patient was treated with topical ganciclovir eye gel (5 times daily), dexamethasone phosphate (0.1%, 3 times daily), and dorzolamide-timolol maleate eyedrops (2 times daily), combined with systemic acetazolamide (125 mg, 3 times daily) and valaciclovir treatment (1000 mg, 3 times daily). Clinical improvement with reduced intraocular pressure was observed within 1 week. Six weeks post-DMEK, the patient was referred to the Rotterdam Eye Hospital (Rotterdam, The Netherlands) for a second opinion. On presentation, the best-corrected visual acuity of the operated eye was “counting fingers” at 2 m (Snellen Vision 2/60) with an elevated intraocular pressure (24 mmHg). Slit-lamp examination showed mild conjunctival hyperemia, intact but irregular epithelium, diffuse corneal edema with an almost complete attached DMEK lamella, and a fixed and mid-dilated pupil. On retro-illumination, no iris atrophy was observed. Anterior chamber showed no cells or flare, and the recipient’s lens showed slight cataract. Fundoscopy showed a red reflex without details of the fundus. A regimen of slowly tapered steroid therapy with prolonged antiviral prophylaxis was advised. The corneal graft was still functional 2 years later.

**Table 1:** Characteristics of DMEK Patients and Clinical Samples Used for Whole HSV-1 Genome Sequencing

	Patient 1		Patient 2		Patient 3	
	*Info*	*HSV-1*∗	*Info*	*HSV-1*∗	*Info*	*HSV-1*∗
Gender/age	Male/74 yrs		Female/73 yrs		Unknown	
Underlying disease	Fuch’s endothelial dystrophy		Fuch’s endothelial dystrophy		Unknown	
HSV serostatus	IgG positive		IgG positive		Unknown	
						
Donor cornea	Donor A†	Positive	Donor B†	Positive	Donor C†	Positive
						
Post-DMEK disease	Yes		Yes		Unknown	
						
Clinical samples (ID)‡	Cornea remnant (S01)	Ct 19.9	Cornea remnant (S07)	Ct 31.8	Cornea remnant (S11)	Ct 32.0
	Culture medium (S02)	Ct 21.2	Culture medium (S08)	Ct 29.7	Culture medium (S12)	Ct 29.3
	Transport medium (S03)	Ct 30.9	Transport medium (S09)	Ct 23.6		
	Aqueous humor (S04)	Ct 23.9	Aqueous humor (S10)	Ct 17.6		
	Virus culture of #1 (S05)	Ct 16.0				
	Virus culture of #2 (S06)	Ct 15.9				

Ct = cornea tissue; DMEK = Descemet's membrane endothelial keratoplasty; HSV-1 = herpes simplex virus type 1; IgG = immunoglobulin G.

∗ Results of HSV-1 polymerase chain reaction (PCR) shown as positive or negative. Cornea tissue values of positive samples are provided.

† Donor ID of donor cornea tissue.

**Figure 1:**
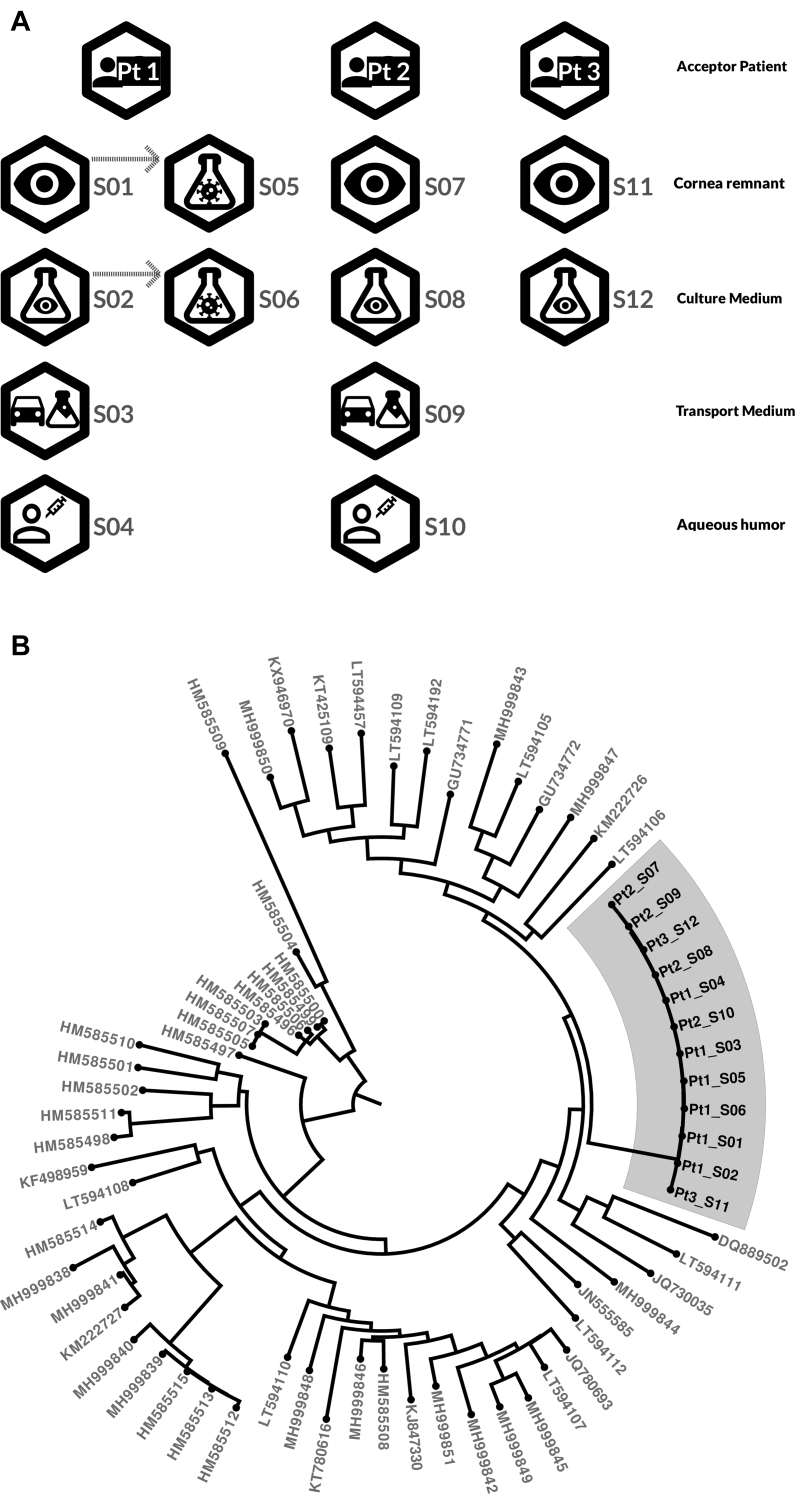
Phylogenetic tree. **A,** Schematic representation of samples sequenced in this study, indicating sample source and patient. Further details for each sample are shown in Table 1. **B,** Maximum likelihood phylogenetic tree of herpes simplex virus type 1 (HSV-1) genomes from the clinical specimens in this study (shaded area) together with available GenBank reference isolates (Supplementary Table 1). In a circular phylogeny such as this, the distance between the center of the tree and each of the samples at the edge represents genetic similarity of the samples being compared. Internal bifurcations in the tree represent inferred common ancestors to the samples they lead to. Samples in this study form a single cluster with small genetic distances between the samples and a large distance to the closest sample not part of the study. Furthermore, the 3 patients in this study do not form separate clusters, supporting a common origin for all 12 HSV-1 DNA-positive clinical samples sequenced in this study. The high similarity of the samples is further detailed in the network analysis shown in Figure 2.

**Figure 2:**
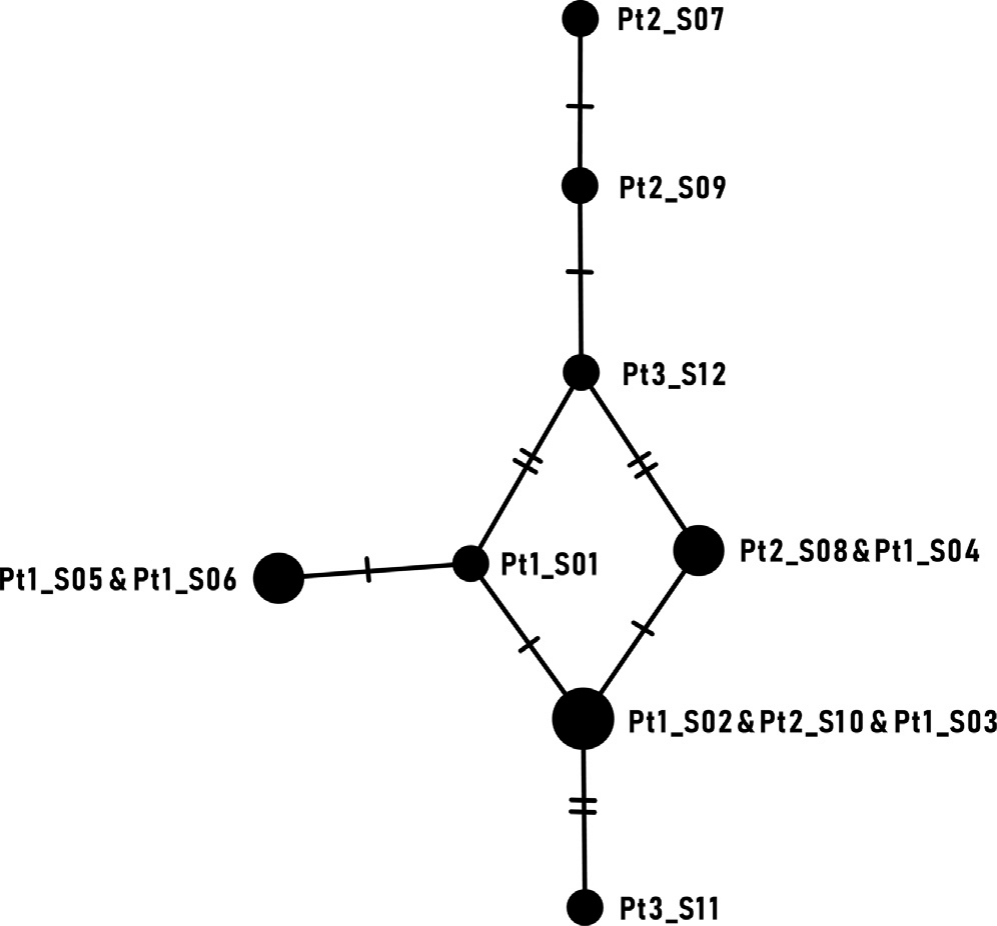
Population analysis with reticulate trees analysis. To allow a close inspection of the genetic distances between the samples in this study, we performed a minimum spanning network analysis from these study samples. In these analyses, groups of related samples form tightly knit networks. The number of nucleotides that are different between each pair of connected samples correspond to the shortest distance possible to connect all 12 HSV-1 DNA-positive clinical samples sequenced in this study. The number of nucleotides at each link are denoted by hatch marks. Samples that are genetically identical at consensus level were merged into a single node. Node size is proportional to the number of samples it represents. We observed no segregation of the samples from the different patients into separate groups. Further analyses on pairwise differences between samples are available in Supplementary Figures 1 and 2.

‡ Numbers refer to HSV-1 genomes obtained from the respective clinical specimens and subsequently used in Figures 1 and 2 (see also Table S1).

Patient 2, a 73-year-old woman with a history of FECD, underwent DMEK surgery in her left eye on the same day in the same cornea clinic with donor tissue from the same eye bank as patient 1. At day 1 post-DMEK, she developed complete corneal erosion followed 2 days later by development of KPs and elevated intraocular pressure. The KPs were nonresponsive to steroids. No signs of bacterial or fungal infection were detected, and endophthalmitis was not suspected clinically. Herpes simplex virus type 1 DNA was detected in a diagnostic AC tap obtained 10 days post-DMEK (Table 1). The patient received the same medication as patient 1 and was also referred to Rotterdam Eye Hospital at 6 weeks post-DMEK for a second opinion. At presentation, visual acuity was 1/60 in the operated eye with an elevated intraocular pressure of 28 mmHg. The anterior segment showed signs of mild conjunctival hyperemia, diffuse corneal edema with a complete attached DMEK lamella, and a fixed and dilated pupil. Retro-illumination revealed no iris atrophy, and on fundoscopy only a red fundus reflex was visible. Ultrasound examination showed a normal posterior segment. Four months post-DMEK, recurrent herpetic keratitis occurred and approximately 1 year later recurrent episodes of elevated intraocular pressure. The operated cornea completely decompensated with deep corneal edema and scarring, necessitating PKP and a glaucoma shunt to improve vision and alleviate pain. Real-time PCR analyses on material obtained during the penetrating keratoplasty, both AC tap and removed cornea button, were HSV-1 DNA negative. Histology of the removed corneal button showed complete atrophy of the endothelium and increased cellularity indicating chronic keratitis (data not shown).

### Diagnostic Virology

Surplus cornea donor and recipient samples were sent by the eye bank to the Viroscience laboratory (Erasmus MC; Rotterdam, The Netherlands) to determine if the ocular HSV-1 complications were due to reactivation of latent recipient’s virus (both recipients were HSV-1 immunoglobulin-G positive at the time of DMEK; Table 1) or graft-to-host HSV-1 transmission.^9^, ^10^, ^11^, ^12^, ^13^ The sample set included corneal remnants from donors A and B used to prepare DMEK grafts for patients 1 and 2, respectively, conditioned culture media in which the donor corneas were cultured for several days, and media used to transport the prepared DMEK grafts to the operating room. In addition, the set contained samples (cornea remnant, culture, and transport medium) from another donor (donor C) from whom the DMEK graft was prepared on the same day as for patients 1 and 2, and cornea remnants from 6 randomly selected donors that were processed in the same period as donors A to C. No clinical information is available from recipients of cornea tissues of donor C and the 6 randomly selected donors.

Cornea remnants of donors A and B used for DMEK on patients 1 and 2 tested HSV-1 DNA^POS^. Notably, infectious HSV-1 was isolated by cell culture from the HSV-1 DNA^POS^ culture medium and cornea remnant of donor A. Samples from donors B and C were also subjected to virus culture, but no infectious virus was isolated (data not shown). Cornea remnants from donor C were also HSV-1 DNA^POS^, but no clinical information or specimens were available from recipient of this graft. No HSV-1 DNA was detected in cornea remnants of the 6 randomly selected donors processed by the same eye bank in the same period (data not shown).

### Whole HSV-1 Genome Sequencing

Viral genomes of HSV-1 DNA^POS^ specimens of the respective donors and recipients were recovered by target-enriched deep sequencing to determine the origin of the inciting HSV-1 (Table 1).^16^^,^^17^ Sequencing data were filtered for low quality and human reads, and mapped using a combination of de novo and reference-based consensus (HSV-1 GenBank ID: NC_001806.2).^13^ The percent genome coverage correlated with RT-PCR cycle threshold values of the respective samples (Pearson correlation test, *r*^*2*^ = 0.94, *P* < 0.0001) (Supplemental Table 1). Infectious HSV-1 isolated by cell culture from donor A specimens was used as technical positive control for viral relatedness. For context and negative control, a set of 57 unrelated HSV-1 sequences were retrieved from GenBank (release 242.0) (Supplemental Table 2). All 12 samples sequenced had a high degree of genetic similarity, forming a distinct cluster (Fig 1). Notably, sample pairs from the same patient have similar genetic distances to sample pairs among all 3 patients (Wilcoxon rank-sum test, *P* > 0.05), and no evidence for recombination between samples was found (Phi test, *P* = 0.85) (Supplementary Fig 1). De novo assembly suggested larger pairwise differences than reference-based analyses, with both data sets within the same order of magnitude as the positive controls and a 10-fold lower genetic distance than natural HSV-1 diversity found when looking at unrelated sequences (Supplementary Fig 2).

To determine relationships between the HSV-1 genomes, a minimum spanning network was constructed using Population Analysis with Reticulate Trees.^27^ Figure 2 shows how samples are related to one another with number of nucleotide differences between samples indicated. Notably, several samples from patients 1 and 2 were identical. The small numbers of nucleotide substitutions at consensus level are of the same order as those expected from sequencing artefacts.^29^ Overall, the data demonstrate a recent common ancestor between all sequenced samples, indicating cross-contamination and subsequently a cluster of graft-to-host HSV-1 transmission in this cornea transplant setting.

## Discussion

Descemet's membrane endothelial keratoplasty, first described by Melles et al in 2006,^4^ has developed into a common therapeutic intervention to restore corneal clarity in patients with endothelial disorders worldwide.^1^ With this technique, more than 1 graft can be prepared from 1 donor cornea to restore sight successfully in multiple patients.^2^^,^^5^^,^^6^ Despite its benefits, DMEK is technically more challenging than conventional PKP for both eye banks and surgeons in graft preparation, storage, and transplantation, which pose a higher risk of microbial (cross-)contamination.^1^^,^^3^^,^^5^^,^^6^ Infectious complications after DMEK are considered uncommon, but this may be underestimated because of the atypical presentation of ocular disease at the lamellar interface of recipient and donor tissue when an infected lamellar graft is transplanted via the AC route.^8^, ^9^, ^10^ The anterior chamber is an immune-privileged site, and introduction of microorganisms directly into the AC via DMEK may lead to atypical clinical presentations due to selective downregulation of both local and systemic immunity.^30^^,^^31^ We report on 2 FECD patients who developed atypical ocular complications within a few days after DMEK. Both patients presented with high intraocular pressure, localized corneal edema with whitish KPs, and minimal AC inflammation that simulated early graft rejection.^6^^,^^9^^,^^32^ Etiology of corneal endotheliitis is extensive and may be systemic, therapeutic, or pathogen related.^9^^,^^32^ The rapid onset of disease in otherwise healthy immunocompetent individuals undergoing an uneventful DMEK precluded systemic and therapeutic etiology. Moreover, unresponsiveness to steroid treatment excluded early graft rejection.^2^^,^^4^^,^^5^ In the absence of evidence for bacteria or fungi detection, viral etiology was suspected and identified as HSV-1 by RT-PCR on AC taps of both patients.^8^, ^9^, ^10^

Herpetic infection in the context of corneal grafting has a variable presentation that may manifest as graft edema, epithelial defects, anterior chamber reaction, graft-host infiltrates, or raised intraocular pressure.^7^^,^^9^^,^^10^^,^^12^^,^^13^ A definitive diagnosis of herpetic infection in lamellar grafts may be challenging to establish because the clinical signs overlap with those of graft rejection. Indeed, similar clinical signs have been described earlier in case of HSV-1 reactivation, but occurred relatively late after lamellar keratoplasty.^33^, ^34^, ^35^

Comprehensive RT-PCR analysis and virus culture of both recipient and donor specimens, and the patient’s responsiveness to antiviral therapy, suggested symptomatic graft-to-host HSV-1 transmission, potentially of shared origin in all 3 DMEK recipients described. Indeed, whole genome sequencing (WGS) of surplus HSV-1 DNA^POS^ recipient and donor specimens demonstrated a high similarity between 12 HSV-1 isolates of the 3 DMEK patients. The combined data indicate cross-contamination of donor cornea tissues, most likely at the respective eye bank, leading to transplantation of HSV-1–infected DMEK grafts and subsequent symptomatic anterior herpetic disease in 2 recipients. Source of infectious HSV-1 may be a donor cornea shedding HSV-1 asymptomatically at the time of enucleation or reactivation of latent cornea-derived virus that remained unnoticed during processing and culture of the respective donor cornea tissues.^6^^,^^9^^,^^10^^,^^35^ Alternatively, but less likely, is contamination of the cultures by a co-worker of the respective eye bank who shed HSV-1 at the time of tissue preparation.

With an increasing world population and better access to health care, the demand of cornea transplantations will increase, leading to challenges not only in the availability of sufficient donor tissues but also in the need for optimal preservation and successful surgical use and follow-up of these scarce tissues. Descemet's membrane endothelial keratoplasty has revolutionized the management of corneal endothelial failure by largely meeting these demands, but with the growing number of surgeries performed worldwide, unprecedented complications are to be expected.^1^^,^^3^^,^^5^ Although the majority are due to technical failures, immune reactions, or pre-existing herpetic eye disease,^36^ vigilance is warranted to be aware of herpesvirus infections in cornea donor tissues both before and after DMEK where clinical signs may closely resemble graft rejection.^6^^,^^9^^,^^10^^,^^35^ Our study underpins this notion by demonstrating that cross-contamination and subsequent graft-to-host HSV-1 transmission by DMEK can lead to atypical sight-threatening herpetic eye disease, a disease that can easily be prevented by taking appropriate technical and clinical measures at both eye bank and surgical levels.

We propose 4 solutions to prevent HSV-1 transmission by DMEK procedures. First, most important is to inform ophthalmologists that complications shortly after DMEK procedure can be caused by HSV-1, that the clinical picture may be atypical for ocular HSV-1 infection, and that the infection can be due to reactivation of endogenous latent HSV-1 or donor-derived acquired following the DMEK procedure. Thus, in the event that a complication occurs shortly after DMEK, molecular diagnostics for HSV-1 should be performed (e.g., polymerase chain reaction [PCR]), and if positive, the respective cornea surgeon and eye bank should be informed to initiate prompt antiviral therapy and to update procedures to prevent such transmissions in the future, respectively. Second, HSV-1 replication in donor corneas during the culture period at the eye bank can occur without cellular changes in the graft.^37^^,^^38^ Thus, microscopic examination performed before delivery by the eye bank may miss an ongoing HSV-1 infection in the graft. Therefore, we propose to perform HSV-1 PCRs on donor cornea culture media before its scheduled release for transplantation, and if positive, reject the donor graft. The high sensitivity and specificity of diagnostic HSV-1 PCRs allow for application of this technique in settings where the pretest probability of HSV-1 is low, such as in eye banks.^14^ Third, HSV-1 antivirals such as acyclovir can be added to the culture medium of the donor cornea. It should be noted that these antivirals only inhibit replication of HSV-1 but do not inactivate virus particles, because acyclovir is not virucidal.^39^ Future work is warranted to determine the effect of antivirals on the quality of cornea grafts. Finally, exclude donors with facial lesions suspect for active HSV-1 infections such as herpes labialis.
