# Supplementary material for: Cluster of Symptomatic Graft-to-Host Transmission of Herpes Simplex Virus Type 1 in an Endothelial Keratoplasty Setting
Source: Ophthalmol Sci. 2021 Aug 12;1(3):100051. doi: 10.1016/j.xops.2021.100051 (PMC9562293; doi:10.1016/j.xops.2021.100051)
Supplement: Supplementary Fig 1 [file mmc1.pdf]

Supplementary Figure 1

A

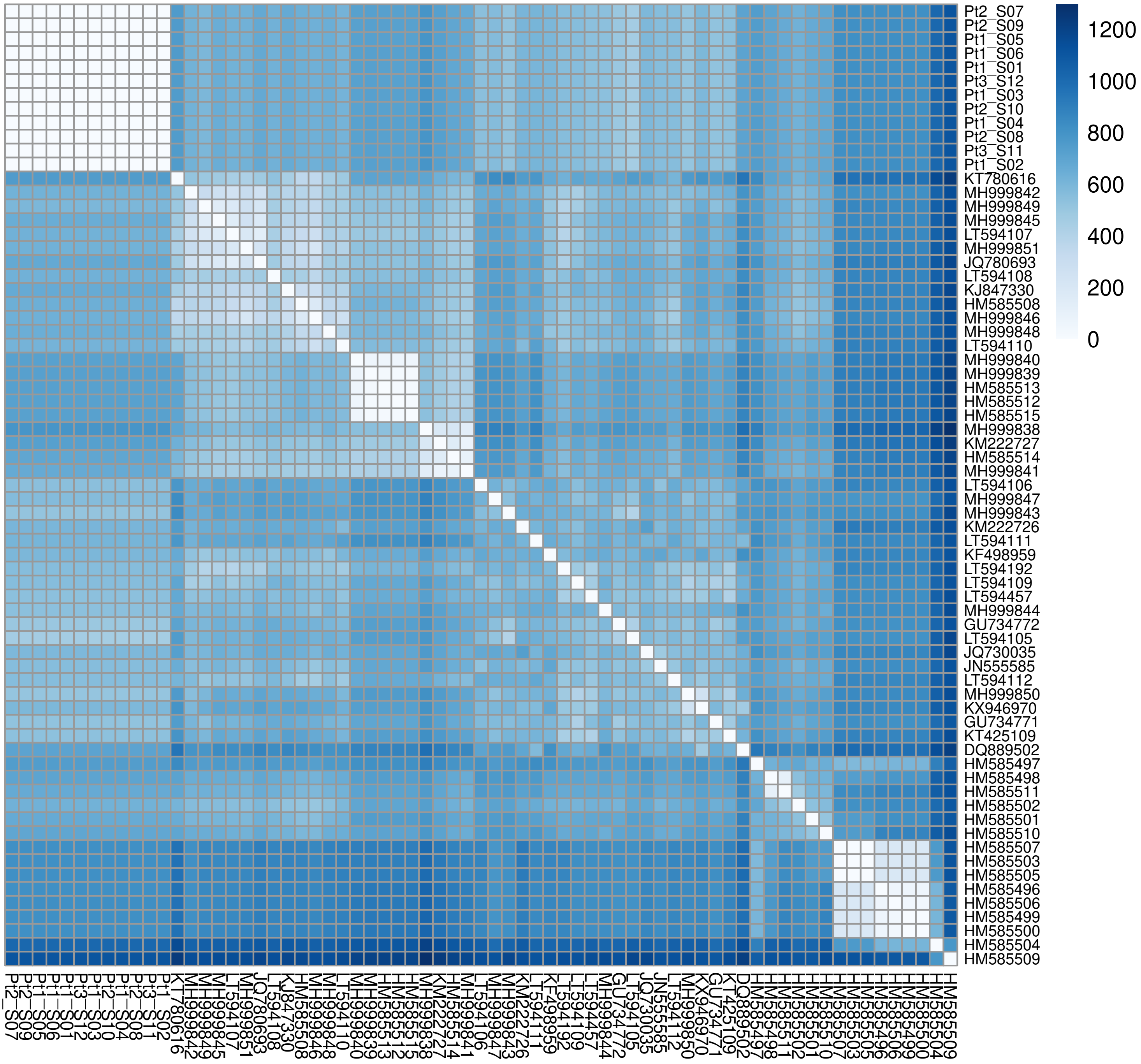

B

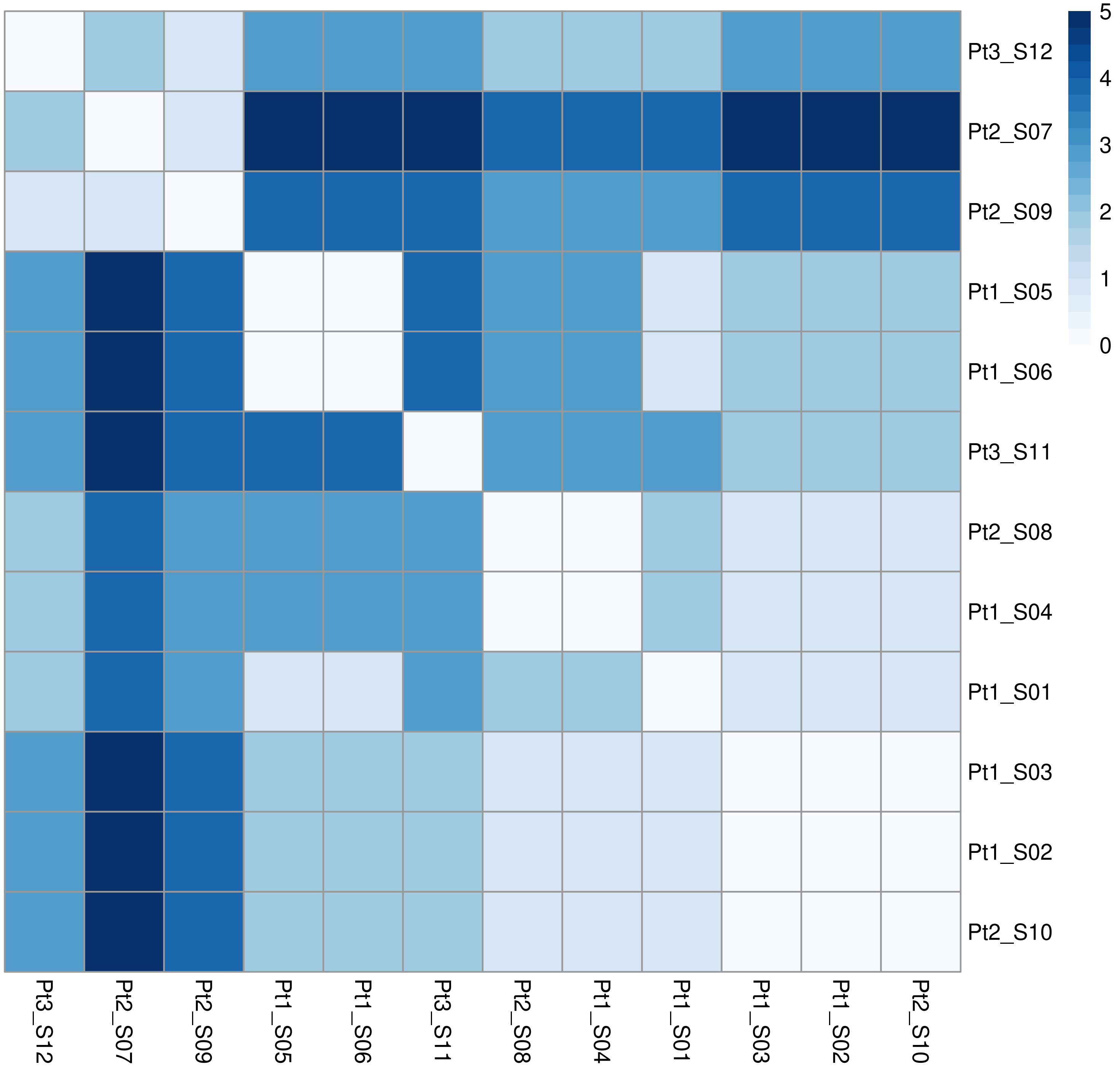

Supplementary Figure 1: This figure containing heatmaps representing pairwise genetic distances between consensus sequences, expands the analysis represented in Figures 1 and 2 by including not just the shortest and most likely path connecting every two samples, but the pairwise genetic distances between all samples under comparison. A) includes the 12 samples we describe, forming a cluster of small genetic distances in the upper left corner of the heatmap, as well as consensus sequences retrieved from GenBank to represent previously sampled HSV-1 genetic diversity. B) includes just the 12 HSV-1 DNA positive clinical samples sequenced in this study with an appropriate colour scale for the small number of differences observed. Scales range from low number of differences (white) to high number of differences (dark blue). Samples are ordered by hierarchical clustering, further highlighting the absence of clustering by patient within this study. Overall, the small numbers of substitutions at consensus level within our samples, which seem to correlate with obtained sequenced depth, is of the same order as that expected from minor sequencing artefacts.
