# Supplementary material for: Cluster of Symptomatic Graft-to-Host Transmission of Herpes Simplex Virus Type 1 in an Endothelial Keratoplasty Setting
Source: Ophthalmol Sci. 2021 Aug 12;1(3):100051. doi: 10.1016/j.xops.2021.100051 (PMC9562293; doi:10.1016/j.xops.2021.100051)
Supplement: Supplementary Fig 2 [file mmc2.pdf]

Supplementary Figure 2

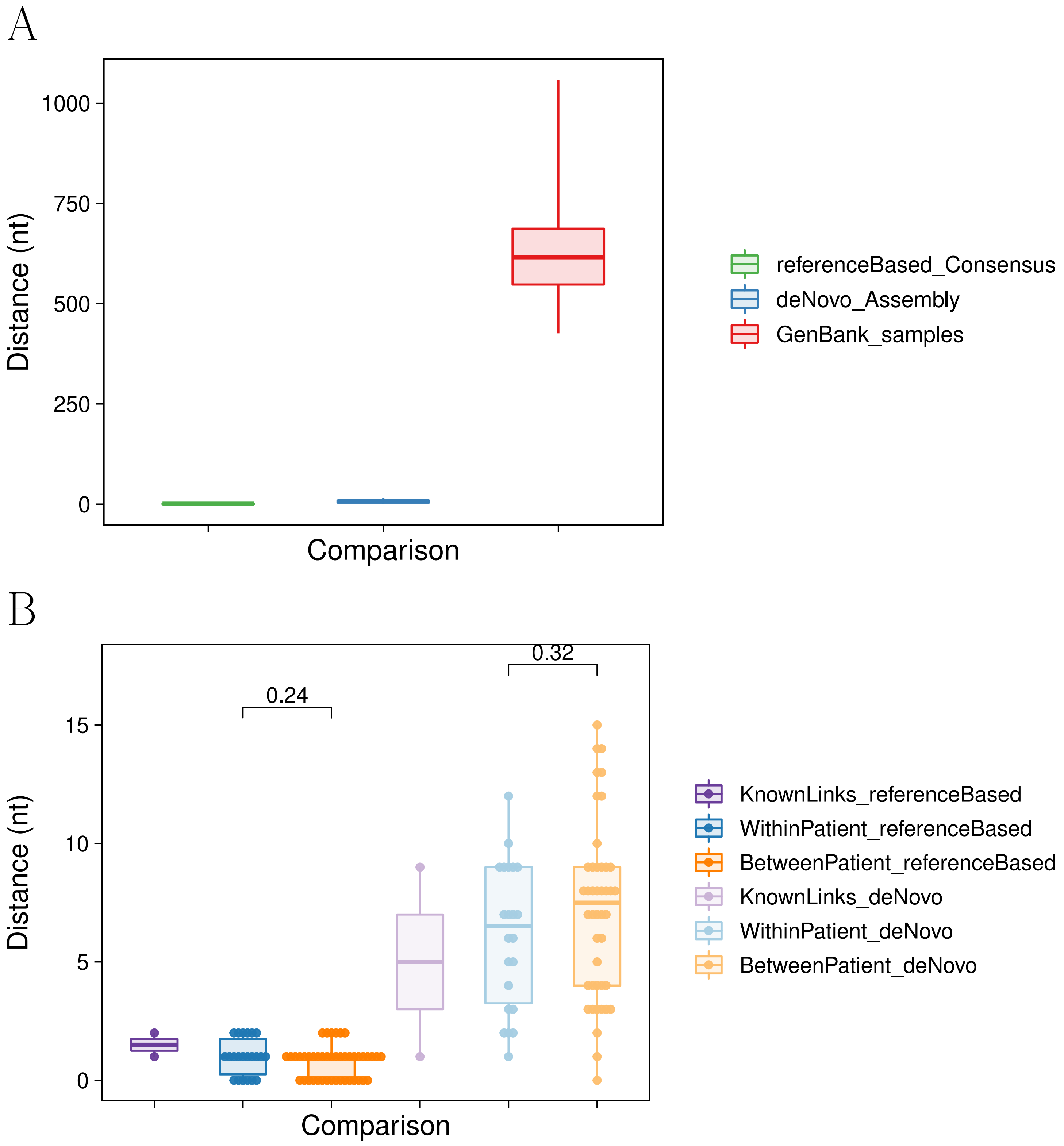

Supplementary Figure 2: Different analysis methodologies can produce slightly different results from the same sequencing data. To demonstrate the robustness of our conclusions to analytical choices, we process the data using two different analysis approaches: referenced based consensus building and de novo assembly. This figure illustrates the distances observed for the sequences of dine with each of the methods compared among themselves and with publicly available GenBank consensuses. A) Boxplots from pairwise nucleotide (nt) distances between samples within this dataset when processed using reference-based consensus building (green), de novo assembly (blue) and between the 12 clinical samples in this study and existing unrelated samples retrieved from GenBank (red). These data illustrate how similar the samples within this dataset are, regardless of methodology used, when compared to the natural variability observed for different HSV-1 isolates previously sequenced. B) Boxplots of pairwise distances between samples of this data set by analysis type and relationship between the samples being compared. Pairwise distances between samples and their

respective viral culture are shown in purple for reference-based consensus and lilac for de novo assembly. Pairs of samples from the same donor/patient pair are shown in blue and light-blue, and pairs of samples between patients, are shown in orange and yellow, respectively. If patient samples are of different origin, one would expect a lower distance when looking at within patient comparisons compared to when looking at between patient comparisons. In this case, Mann-Whitney-Wilcoxon analysis comparing the within patient sample distances with between patient sample distances, for each analysis methodology are consistent and do not find a statistically significant difference.
