# Supplementary material for: Cluster of Symptomatic Graft-to-Host Transmission of Herpes Simplex Virus Type 1 in an Endothelial Keratoplasty Setting
Source: Ophthalmol Sci. 2021 Aug 12;1(3):100051. doi: 10.1016/j.xops.2021.100051 (PMC9562293; doi:10.1016/j.xops.2021.100051)
Supplement: Supplemental Table 1 [file mmc3.pdf]

Supplementary Table 1

ENA Run	ENA Experiment	ENA Filename	Patient	Sample	Ct	Num Reads	Num Mapped	%OTR	Coverage%	Depth (w/dup)	Depth (wo/dup)
ERR5236833	ERX5039984	SampleS1_PatientPt1	Pt1	S01	21.8	1587357	1557399	98.11%	98	2,459.66	2,348.99
ERR5236843	ERX5039994	SampleS2_PatientPt1	Pt1	S02	21.2	1855091	1826648	98.47%	98	2,744.03	2,641.52
ERR5236854	ERX5040005	SampleS3_PatientPt1	Pt1	S03	30.9	422751	402281	95.16%	98	1,590.68	585.00
ERR5236876	ERX5040027	SampleS4_PatientPt1	Pt1	S04	23.9	1975431	1940338	98.22%	99	2,857.43	2,752.99
ERR5236731	ERX5039882	SampleC1_PatientPt1	Pt1	S05	16	3382634	3335950	98.62%	99	4,355.93	4,233.46
ERR5236828	ERX5039979	SampleC2_PatientPt1	Pt1	S06	15.9	2486305	2452233	98.63%	99	3,192.59	3,091.83
ERR5236835	ERX5039986	SampleS1_PatientPt2	Pt2	S07	31.8	39527	34114	86.31%	90	457.67	49.65
ERR5236847	ERX5039998	SampleS2_PatientPt2	Pt2	S08	29.7	208766	191564	91.76%	97	1,411.21	278.83
ERR5236857	ERX5040008	SampleS3_PatientPt2	Pt2	S09	32.6	90381	77527	85.78%	95	620.49	99.07
ERR5236885	ERX5040036	SampleS4_PatientPt2	Pt2	S10	17.6	2830735	2790866	98.59%	99	3,794.25	3,683.54
ERR5236837	ERX5039988	SampleS1_PatientPt3	Pt3	S11	32	32857	27803	84.62%	88	363.56	40.93
ERR5236850	ERX5040001	SampleS2_PatientPt3	Pt3	S12	29.3	107893	93875	87.01%	96	1,048.35	139.77

Supplementary Table 1: The data sequenced in this study has been deposited under project accession PRJEB42849. The following table provides correspondence between each of the identifiers associated with the data (fields starting with ENA) and the patient and sample number that is used throughout the manuscript to refer to them. Besides the annotation, we also provide quality control metrics for these 12 samples including: Ct value as a proxy for viral load (Ct); total number of reads sequenced for the sample (Num Reads); number of reads that mapped to the reference genome (Num Mapped); the percentage of on target reads (%OTR); the percentage of the reference genome that is covered by sequencing data (Coverage%); average read depth when including likely PCR duplicate reads (Depth (w/dup)); average read depth when likely PCR duplicates are removed (Depth (wo/dup)).
