# Supplementary material for: Cluster of Symptomatic Graft-to-Host Transmission of Herpes Simplex Virus Type 1 in an Endothelial Keratoplasty Setting
Source: Ophthalmol Sci. 2021 Aug 12;1(3):100051. doi: 10.1016/j.xops.2021.100051 (PMC9562293; doi:10.1016/j.xops.2021.100051)
Supplement: Supplemental Table 2 [file mmc4.pdf]

Supplementary Table 2

GenBank ID	PubMed ID	DOI
DQ889502	17218138	10.1016/j.micinf.2006.10.019
GU734771	20219902	10.1128/JVI.00312-10
GU734772	20219902	10.1128/JVI.00312-10
HM585496	22417106	10.1111/j.1749-6632.2011.06358.x
HM585497	22417106	10.1111/j.1749-6632.2011.06358.x
HM585498	22417106	10.1111/j.1749-6632.2011.06358.x
HM585499	22417106	10.1111/j.1749-6632.2011.06358.x
HM585500	22417106	10.1111/j.1749-6632.2011.06358.x
HM585501	22417106	10.1111/j.1749-6632.2011.06358.x
HM585502	22417106	10.1111/j.1749-6632.2011.06358.x
HM585503	22417106	10.1111/j.1749-6632.2011.06358.x
HM585504	22417106	10.1111/j.1749-6632.2011.06358.x
HM585505	22417106	10.1111/j.1749-6632.2011.06358.x
HM585506	22417106	10.1111/j.1749-6632.2011.06358.x
HM585507	22417106	10.1111/j.1749-6632.2011.06358.x
HM585508	22417106	10.1111/j.1749-6632.2011.06358.x
HM585509	22417106	10.1111/j.1749-6632.2011.06358.x
HM585510	22417106	10.1111/j.1749-6632.2011.06358.x
HM585511	22417106	10.1111/j.1749-6632.2011.06358.x
HM585512	22417106	10.1111/j.1749-6632.2011.06358.x
HM585513	22417106	10.1111/j.1749-6632.2011.06358.x
HM585514	22417106	10.1111/j.1749-6632.2011.06358.x
HM585515	22417106	10.1111/j.1749-6632.2011.06358.x
KM222726	25827418	10.1128/mBio.02213-14
KM222727	25827418	10.1128/mBio.02213-14
JN555585	22417106	10.1111/j.1749-6632.2011.06358.x
MH999851	Unpublished	
MH999850	Unpublished	
KJ847330	Unpublished	
JQ780693	Unpublished	
KT425109	Unpublished	
MH999838	Unpublished	
MH999839	Unpublished	
MH999841	Unpublished	
MH999840	Unpublished	
JQ730035	23021301	10.1016/j.virol.2012.08.043
MH999847	Unpublished	
MH999842	Unpublished	
MH999844	Unpublished	
MH999845	Unpublished	
MH999843	Unpublished	
MH999846	Unpublished	
MH999848	Unpublished	
MH999849	Unpublished	
LT594105	Unpublished	
LT594106	Unpublished	
LT594107	Unpublished	
LT594108	Unpublished	
LT594109	Unpublished	
LT594110	Unpublished	
LT594111	Unpublished	

GenBank ID	PubMed ID	DOI
LT594112	Unpublished	
LT594192	Unpublished	
LT594457	Unpublished	
KT780616	Unpublished	
KF498959	Unpublished	
KX946970	28126930	10.1128/genomeA.01392-16

Supplementary Table 2: The following table lists the GenBank accession numbers for the samples that were used in this analysis has representatives of the genetic variability observed in previously sequenced HSV-1 genomes. When the information was available in GenBank, we also included the reference for the manuscript where the sample was first described.
